# Examining correlates of aggression and mediating effect of psychological distress between exposure to media violence and aggression in lebanese adults

**DOI:** 10.1186/s40359-023-01232-0

**Published:** 2023-06-29

**Authors:** Alfred Chabbouh, Souheil Hallit, Nour Farah, Christina Youssef, Abdo Hankache, Feten Fekih-Romdhane, Zeinab Bitar, Sahar Obeid

**Affiliations:** 1 Faculty of Medical Sciences, Lebanese University, Beirut, Lebanon; 2 School of Medicine and Medical Sciences, Holy Spirit University of Kaslik, Jounieh, P.O. Box 446, Lebanon; 3 Applied Science Research Center, Applied Science Private University, Amman, Jordan; 4 Research Department, Psychiatric Hospital of the Cross, Jal Eddib, Lebanon; 5 The Tunisian Center of Early Intervention in Psychosis, Department of Psychiatry “Ibn Omrane”, Razi Hospital, Manouba, 2010 Tunisia; 6 Faculty of Medicine of Tunis, Tunis El Manar University, Tunis, Tunisia; 7 Faculty of Medicine, Paris-Saclay University, Le Kremlin-Bicêtre, France; 8 Social and Education Sciences Department, School of Arts and Sciences, Lebanese American University, Jbeil, Lebanon

**Keywords:** Media violence, Aggression, Psychological distress, Lebanon

## Abstract

**Background:**

Violent media is the most consumed type of media in Lebanon. Many studies have linked exposure to media violence to increased aggression and psychological distress. As Lebanon is going through socio-political turmoil, we aimed to [1] explore the correlates of aggression (i.e., sociodemographic factors, BMI, loneliness, social competence, and psychological distress) in a sample of Lebanese adults from the general population, and [2] to examine the mediating effect of psychological distress in the association between exposure to media violence and aggression in this sample.

**Methodology:**

Adults were recruited through online convenience sampling. We employed scales to assess content-based media exposure (C-ME), aggression (BPAQ-SF), psychological distress (DASS-8), loneliness (JGLS), and perceived social competence (PSCS).

**Results:**

Exposure to media violence was associated with all four aggression subtypes (verbal, physical, hostility, and anger). Psychological distress partially mediated all these associations; higher exposure to media violence was significantly associated with more psychological distress, which was significantly associated with higher levels of all types of aggression. Moreover, higher exposure to media violence was significantly associated with higher levels of all types of aggression.

**Conclusion:**

In the sociopolitical context of Lebanon, violent media could be considered a public hazard. Psychological distress likely potentiates the association between exposure to violent media and aggression. Future research should focus on determining what components of psychological distress underpin this mediation.

## Introduction

Media has become an integral part of human life, more important than clean water in the eyes of some [1]. New media devices, including cellular and online technologies, have quickly spread in the Middle East over the last years. The number of cell phone owners and Internet users has grown rapidly in the region [2]. According to Internet World Stats, the estimated number of internet users in Lebanon corresponds to an internet penetration rate of 81.8% [1], an estimate that has been steadily increasing [3]. This unprecedented media consumption has raised concerns about the impact of social media use on mental health and well-being in Lebanon, particularly in the context of the economic and political instability that the country is facing in recent years [4].

Most research on the effects of media on mental health focuses on the quantity of general media exposure [1]. However, different types of media content can have different impacts. For instance, unlike educational television, early exposure to entertainment television including violent ones predicted attentional problems in children 5 years later [5]. Moreover, media related to drug use are associated with increased willingness of adolescents to partake in these behaviors [1, 6, 7]. In a world where media is omnipresent, the question of what media is consumed is becoming far more important than how much is consumed [1].

One particularly important media type is violent media. Media violence is defined as visual portrayals of acts of physical aggression by one human against another [8]. Exposure to media violence is distinct from active aggressive behaviors towards someone through electronic forms of contact (e.g. cyberbullying) [9]. Portrayals of violence in the media are controversial with violence sometimes being pictured as socially admissible, tolerable, and a common method to manage conflicts. Violent media can originate from television and films, real-life news, and video games. All aforementioned media types are associated with aggression as it seems the media content characteristics per se (e.g. presentation, justifiability, consequences, realism, etc.), rather than the source of media, are the major determinants of the risk of violence [8].

### The relationship between exposure to media violence and aggression

Aggression is an umbrella term for different psychological processes. Studies have consistently found a four-factor model in tools assessing aggression [10, 11] which they operationalized into four subtraits [12]. The first two are goal-directed behavioral components involving harming others: physical aggression and verbal aggression. The third subtrait is hostility, a cognitive component relating to injustice and ill will. The final component is anger, which is the affective nature of aggression. Due to its higher correlation with other components, anger has been proposed as the bridge between hostility (i.e. aggressive thoughts) and the behavioral components.

Most reviews and meta-analyses have reported a strong relationship between media violence and the likelihood of aggressive behavior [13–16]. Repeated exposure to media violence has been proposed to dampen aversive physiological responses of individuals, leading to a process of desensitization. This desensitization, in turn, impacts not just to screen violence but also, real-world violence [17]. Coupled with other psychological processes, media violence became a catalyst towards aggression [18]. However, some researchers disagree on whether current evidence supports the fact that media violence exposure is a risk factor for aggression [19].

Research on the association between media violence and aggression has been especially studied in children. Short-term and long-term processes have been implicated in the effects of media violence on aggression. Short-term effects include priming, arousal, and mimicry, while long-term effects involve learning and desensitization [18]. In fact, media violence can be construed as a possible public health issue, notably affecting the young. Children exposed to violent media are more likely to act aggressively and violently as adults [18]. Additionally, children put through violent media between the ages of 8 and 10 were almost twice as likely to physically abuse their spouses 15 years later [20]. Compared to adults, children are more affected by media violence in the long-term whilst short-term effects seem greater in adults [15]. It is essential to note that the association between media violence and aggressive behavior is not a causal one. Media violence is one of a multitude of factors contributing to aggressive behavior [18]. In the next section, we discuss several other individual and social factors found to be major determinants of violence and aggression.

### Other correlates of aggression

Certain factors are well-established as predictive of aggression, including being young, single, male, and of low socio-economic status [21]. Lower educational attainment is also associated with aggression, likely due to aggressive behaviors themselves [22]. Additionally, the Body Mass Index (BMI) has been an inconsistent predictor of aggression risk. A meta-analysis found higher BMI predicted higher aggression in both girls and boys, although effects were marginally stronger for boys [23]. Another study found that a higher BMI predicted lower arrests [24]. One study found BMI and aggression were only correlated in females [25]. The relationship between BMI and aggressiveness is largely not understood although explanations related to serotonin function, sex hormones, physical attractiveness, and underlying psychosocial determinants have been proposed [23–25]. Psychosocial factors are also implicated. For instance, loneliness plays a significant role in the aggression trajectory [26, 27]. Researchers have found a significant association between loneliness and aggression [28], loneliness being a predictor of physical aggression, verbal aggression, anger and hostility [29–31]. Another psychosocial factor implicated in violence is social competence. Social competence, briefly defined, encompasses positive interpersonal interactions and social skills. One meta-analysis found that less social competence is associated with behavioral problems in children, including aggression [32]. Moreover, in populations with antisocial behaviors, media violence predicted aggressive outcomes [33]. Finally, higher physical activity seems to lower aggression, likely through stress and tension release [34]. This was also observed in children [35].

In addition, psychological distress has been linked to aggressive behaviors in susceptible individuals [36–38]. Psychological distress The association between psychological distress and aggression has been proposed to be likely due to common neuronal circuits relating to emotional regulation and social behaviors [39]. While the absolute risk of violence is low, especially in women, one study found that those with depression were more at risk for violent crimes, notably in certain subgroups [40]. Depression was found to be an independent risk factor for aggression and violence in both children and adults [40, 41]. Moreover, while different psychopathologies attribute different risk weights, all psychopathologies attribute a risk [42]. Hence, psychological distress, the core product of psychopathologies, seems to underpin a higher tendency for aggression.

### Psychological distress as a possible mediator between exposure to media violence and aggression

Based on previous observations that violent media itself seems to contribute to psychological distress on one hand (e.g., [43, 44]), and the well-known association between distress and aggression on the other hand (e.g., [45]), we propose in this paper to test the mediating effect of this factor in the path from media violence exposure to aggression. Some experimental studies found that violent media raised stress levels in participants [46, 47]. In the long-term, daily violent video game playing was associated with depressive outcomes [48]. Moreover, recurrent violent media exposure was shown to fuel a cycle of psychological distress, notably in those who were subject to traumatic events [49]. In sum, violent media is associated with psychological distress in the short-term and long-term [13, 46, 47, 49] and psychological distress is an important but insufficient factor in the development of aggression [21, 42]. This opens up the question on the role of psychological distress in the very well established relationship between violent media and aggression [8]. In fact, stress, one of the three components of psychological distress, was proposed as a mediator between violent video games and aggression [46]. This type of association could possibly reflect a similar mechanism involving psychological distress as mediator between exposure to violent media and aggression. Nevertheless, studies seem to be inconsistent regarding the associations between media effects, aggressive behaviors, and mental health [50]. Factoring in available evidence, we found it valuable to explore whether media violence leads to aggression through a mediation effect by psychological distress, notably in the current context of Lebanon.

### Rationale of the present study

The majority of media consumed by Lebanese people is violent [51]. From bombings to wars, Lebanese people have been exposed to violence for a very long time [52, 53]. This culminated with the Beirut Blast on the 4th of August 2020, in a time when the country was already facing civil unrest, an ongoing socio-economic crisis, and the COVID-19 pandemic. As a result of these factors, Lebanon reported an increase in rates of loneliness [54], desensitization to the suffering of others [55], alexithymia, depression [56], and aggressive behavior [57] during recent years. Violence rates in schools and streets have known an unprecedented increase, and Lebanese youth have gone so far as to be engaged in armed conflicts [58, 59]. Citizens are starting to call violence the new “norm” [60]. When it comes to media specifically, the Lebanese population is familiar with violent images of gunfire, street battles, explosions, missiles, and more [61, 62]. As such, and since no research is available on the matter to date, we aimed to: [1] explore the correlates of aggression (i.e., sociodemographic factors, BMI, loneliness, social competence, and psychological distress) in a sample of Lebanese adults from the general population, and [2] to examine the mediating effect of psychological distress in the association between exposure to media violence and aggression in this sample. We hypothesized that aggression will be positively correlated with BMI, loneliness, and psychological distress, and inversely correlated with perceived social competence. We also expected that psychological distress will mediate the association between exposure to media violence and aggression.

## Methods

### Study design

This study is cross-sectional, conducted between February 2022 and December 2022, which enrolled 403 Lebanese adult participants, all aged above 18 years old. Participants were recruited through convenience sampling; an online link to the survey via the “WhatsApp” application preceded by an introduction containing the information around the purpose of the study, data anonymity, and voluntariness to consent. Participants were encouraged to forward the link to others (family members, friends, etc.) employing the snowball technique.

### Ethical approval

The study proposal was approved by the ethics committee of the School of Pharmacy at the Lebanese International University (2021RC-049-LIUSOP). Submitting the form online was considered equivalent to obtaining a written informed consent.

### Minimal sample size

We used the G*Power software to determine the sample size. The minimum required sample size was 371 participants, considering an alpha error of 5%, a power of 80%, a minimal model R^2^ of 5% and allowing 15 predictors to be included in the model.

### Questionnaire

The questionnaire was divided into two main parts. The first part included the socio-demographic characteristics of the participants (age, sex, marital status, education level, self-reported weight and height, time spent using the mobile phone and Household Crowding Index [HCI]). The latter reflects the socio-economic status of the family and was calculated by dividing the number of people by the number of rooms in the house, excluding the bathrooms and the kitchen [63]. The question about time spent using the mobile phone was based on previous findings [64]. Body Mass Index (BMI) was calculated from self-reported height and weight. Physical activity index was also taken into consideration in our study. The latter was calculated by multiplying the reported intensity, duration, and frequency of daily activity [65]. The digital questionnaire was self-administered in Arabic, the participants’ native language, with approximately 15 min needed to complete it. The second part included the following scales.

**The Content-Based Media Exposure Scale (C-ME)**. The C-ME [1] consists of 17 items measuring the exposure to a broad array of antisocial and risk behavior content in almost all media channels by using 8 items assessing exposure to antisocial media content (e.g., “How often do you watch (on the Internet/TV/games/mobile phone/DVD) people who use drugs?” and 9 items assessing exposure to neutral media content (e.g., “How often do you watch (on the Internet/TV/games/mobile phone/DVD) cooking shows?”). All items are scored using from 1 (never) to 5 (very often). The antisocial media exposure subscale was used in this study to assess exposure to media violence; higher scores indicate higher exposure to media violence (Cronbach’s alpha = 0.90).

**Buss Perry Agression Questionnaire (BPAQ-SF)**. Validated in Arabic [11], the BPAQ-SF [12] consists of 12 items measuring aggression. It includes four subscales: physical aggression (“Given enough provocation, I may hit another person”), verbal aggression (“I can’t help getting into arguments when people disagree with me.”), hostility (“Other people always seem to get the breaks”), and anger (“Sometimes I fly off the handle for no good reason”). Total score for each subscale is the sum or the ratings for its items. Higher scores indicate higher aggressive behavior. Cronbach’s alpha values were as follows: physical aggression (0.73), verbal aggression (0.44), hostility (0.71) and anger (0.74).

**Depression, Anxiety, Stress Scale (DASS-8)**. Validated in Arabic [66], DASS-8 [67] is a reliable and brief instrument used in clinical practice in order to differentiate patients with psychiatric disorders from the general population. The DASS-8 consists of 8 items scored on a 4-point Likert scale with higher total score indicating higher psychopathology (Cronbach’s alpha of 0.91).

**Jong-Gierveld Loneliness Scale**. Validated in Arabic [68], the scale [25] is composed of 5 items assessing loneliness (i.e. “I experience a general sense of emptiness,” “I miss having people around”). Items are scored from 0 to 5 with a higher total score indicating higher loneliness (Cronbach’s alpha of 0.87).

**The Perceived Social Competence Scale (PSCS)** is a brief, 4-item measure of social competence skills and prosocial behavior in children and young adults[69]. Items include, “I help other people” and “I ask others if I can be of help”. The youth respond to each of the items using a Likert-type scale of 1–5 (1 = Not true at all, 5 = Really true) with higher score indicating higher social competence (Cronbach’s alpha = 0.87).

### Translation procedure

The forward-back translation was used for the C-ME and perceived social competence scale using international guidelines [70]. An Arabic native speaker fluent in English did the forward translation, and an English speaker fluent in Arabic unaware of the original and translated scales did the English back-translation. An expert committee composed of health care professionals and a language professional compared the translated versions of the questionnaire with the original ones to check for inconsistencies and to solve any discrepancies between the versions. All ambiguities were resolved after repeating the process of forward-back translation. A pilot study was conducted on 20 persons, with no changes done to any question afterwards.

### Statistical analysis

The SPSS software v.25 was used for statistical analysis. All aggression variables showed a normal distribution (skewness and kurtosis between − 1 and + 1), except for the physical aggression score. The log transformation was applied, which later showed to be normally distributed. The student t test was used to compare two means. The Pearson test was used to correlate two continuous variables. Linear regressions were conducted afterwards, taking each aggression score as a dependent variable. The mediation analysis was conducted using PROCESS MACRO v3.4, model 4, which computed 4 pathways: pathway A from the independent variable to the mediator, pathway B from the mediator to the dependent variable and pathway C and C’ from the independent to the dependent variable reflecting the total and direct effects respectively. Factors that showed a p < .25 in the bivariate analysis were entered as independent variables in each model. P < .05 was considered statistically significant.

## Results

### Sociodemographic **and other characteristics of the participants**

A total of 403 participants enrolled in this study (mean age = 24.56 ± 8.46 years and 73% females). Other characteristics of the sample can be found in Table 1.

**Table 1:** Sociodemographic and other characteristics of the participants (n = 403)

Variable	n (%)
**Sex**	
Male	109 (27.0%)
Female	294 (73.0%)
**Marital status**	
Single	339 (84.1%)
Married	64 (15.9%)
**Education**	
Secondary or less	25 (6.2%)
University	378 (93.8%)
	**Mean ± SD**
Age (in years)	24.56 ± 8.46
Physical activity index	26.81 ± 19.34
Household crowding index (persons/room)	1.09 ± 0.54
Body Mass Index (kg/m^2^)	25.04 ± 14.87
Physical aggression	4.97 ± 2.41
Verbal aggression	6.24 ± 2.44
Anger	6.73 ± 3.09
Hostility	6.75 ± 3.07
Loneliness	12.29 ± 5.07
Perceived social competence	21.90 ± 4.47
Psychological distress	9.21 ± 6.35
Exposure to media violence	44.17 ± 10.35

### Bivariate **analysis**

The bivariate analysis results are shown in Tables 2 and 3. A higher mean physical aggression score was significantly found in males compared to females. More loneliness, psychological distress and exposure to media violence were significantly associated with physical and verbal aggression, hostility and anger. Finally, more perceived social competence was significantly associated with less verbal aggression and anger.

**Table 2:** Bivariate analysis of the categorical variables associated with the aggression scores

Variable	Physical aggression	Verbal aggression	Hostility	Anger
**Sex**				
Male	0.72 ± 0.21	5.95 ± 2.28	6.45 ± 3.09	6.34 ± 2.82
Female	0.63 ± 0.16	6.34 ± 2.49	6.86 ± 3.06	6.87 ± 3.18
*P*	**< 0.001**	0.145	0.237	0.123
**Marital status**				
Single	0.65 ± 0.18	6.15 ± 2.36	6.82 ± 3.08	6.77 ± 3.13
Married	0.67 ± 0.18	6.69 ± 2.80	6.38 ± 3.01	6.50 ± 2.91
*P*	0.379	0.153	0.291	0.518
**Education**				
Secondary or less	0.68 ± 0.22	5.84 ± 2.51	5.96 ± 2.44	5.56 ± 2.40
University	0.65 ± 0.18	6.26 ± 2.44	6.80 ± 3.10	6.81 ± 3.12
*P*	0.495	0.403	0.186	0.051

Numbers in bold indicate significant *p* values

**Table 3:** Bivariate analysis of the continuous variables associated with the aggression scores

Variable	1	2	3	4	5	6	7	8	9	10	11	12
1. Physical aggression	1											
2. Verbal aggression	0.36***	1										
3. Hostility	0.42***	0.44***	1									
4. Anger	0.30***	0.44***	0.57***	1								
5. Loneliness	0.21***	0.29***	0.34***	0.51***	1							
6. Perceived social competence	− 0.09	− 0.13**	− 0.09	− 0.16**	− 0.11*	**1**						
7. Psychological distress	0.23***	0.29***	0.50***	0.57***	0.60***	− 0.09	1					
8. Exposure to media violence	0.23***	0.17**	0.25***	0.25***	0.02	0.06	0.09	1				
9. Age	0.05	0.04	− 0.11*	− 0.07	− 0.07	0.12*	− 0.07	− 0.04	1			
10. Body Mass Index	0.06	0.07	0.09	0.05	− 0.04	0.07	0.01	− 0.03	0.11*	1		
11. Household crowding index	− 0.01	− 0.04	0.02	0.05	0.06	− 0.04	0.14**	− 0.11*	− 0.11*	− 0.07	1	
12. Physical activity index	0.09	− 0.04	0.02	− 0.002	− 0.14**	0.10*	− 0.03	0.10*	0.14**	0.05	− 0.09	1

Numbers in the table reflect Pearson correlation coefficients; *p < .05; **p < .01; ***p < .001

### Multivariable analysis

After adjusting the analysis over all variables that showed a p < .25 in the bivariate analysis, higher exposure to media violence was significantly associated with more physical aggression (Beta = 0.004), verbal aggression (Beta = 0.04), hostility (Beta = 0.06) and anger (Beta = 0.06) (Table 4).

**Table 4:** Multivariable analysis

	Unstandardized Beta	Standardized Beta	*p*	95% CI
**Model 1: Linear regression taking the physical aggression score as the dependent variable (R** ^2^ ** = 0.124).**				
Loneliness	0.01	0.15	**0.001**	0.001; 0.01
Perceived social competence	− 0.004	− 0.09	0.063	− 0.01; 0.001
Psychological distress	0.003	0.11	0.055	− 0.001; 0.01
Exposure to media violence	0.004	0.21	**< 0.001**	0.002; 0.01
Body Mass Index	0.001	0.07	0.121	− 0.001; 0.002
Physical activity	0.001	0.09	0.050	− 0.001; 0.002
**Model 2: Linear regression taking the verbal aggression score as the dependent variable (R**^**2**^ **= 0.148).**				
Loneliness	0.09	0.19	**0.001**	0.04; 0.15
Perceived social competence	− 0.06	− 0.12	**0.014**	− 0.11; − 0.01
Psychological distress	0.06	0.15	**0.010**	0.01; 0.10
Exposure to media violence	0.04	0.16	**0.001**	0.02; 0.06
Body Mass Index	0.01	0.09	0.060	− 0.001; 0.03
**Model 3: Linear regression taking the hostility score as the dependent variable (R**^**2**^ **= 0.312).**				
Loneliness	0.05	0.08	0.111	− 0.01; 0.11
Perceived social competence	− 0.04	− 0.06	0.132	− 0.10; 0.01
Psychological distress	0.20	0.42	**< 0.001**	0.15; 0.25
Exposure to media violence	0.06	0.21	**< 0.001**	0.04; 0.09
Body Mass Index	0.02	0.11	**0.012**	0.01; 0.04
Age	− 0.02	− 0.06	0.152	− 0.05; 0.01
**Model 4: Linear regression taking the anger score as the dependent variable (R**^**2**^ **= 0.418).**				
Loneliness	0.16	0.26	**< 0.001**	0.10; 0.22
Perceived social competence	− 0.07	− 0.11	**0.006**	− 0.13; − 0.02
Psychological distress	0.19	0.38	**< 0.001**	0.14; 0.23
Exposure to media violence	0.06	0.22	**< 0.001**	0.04; 0.09
Age	0.001	0.003	0.933	− 0.03; 0.03

### Mediation analysis

**Fig. 1:**
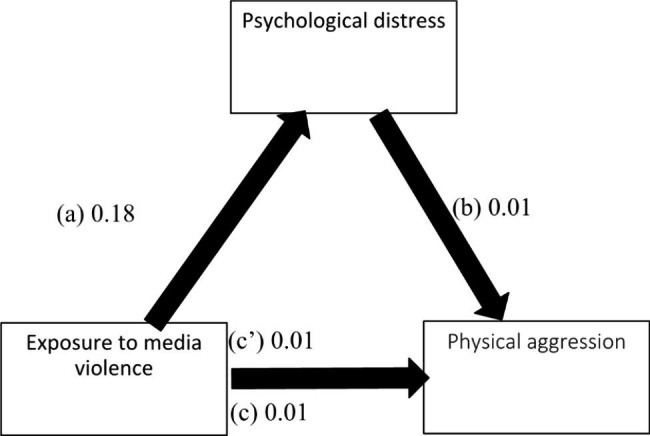
(**a**) Relation between exposure to media violence and psychological distress (R^2^ = .058); (**b**) Relation between psychological distress and physical aggression (R^2^ = .178); (**c**) Total effect of exposure to media violence and physical aggression (R^2^ = .133); (**c**’) Direct effect of exposure to media violence and physical aggression. Numbers are displayed as regression coefficients (standard error). ***p < .001

**Fig. 2:**
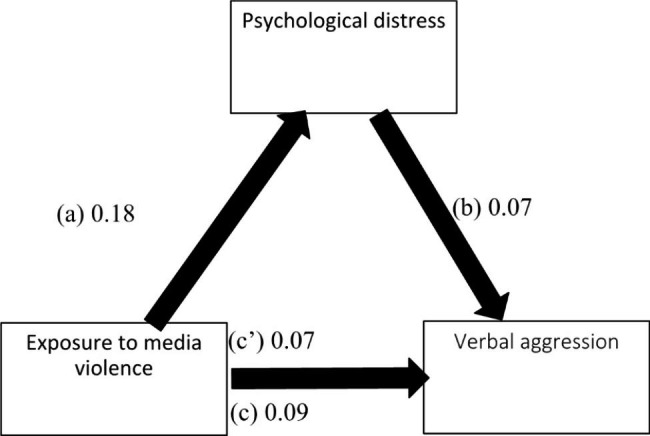
(**a**) Relation between exposure to media violence and psychological distress (R^2^ = .063); (**b**) Relation between psychological distress and verbal aggression (R^2^ = .138); (**c**) Total effect of exposure to media violence and verbal aggression (R^2^ = .079); (c’) Direct effect of exposure to media violence and verbal aggression. Numbers are displayed as regression coefficients (standard error). ***p < .001

**Fig. 3:**
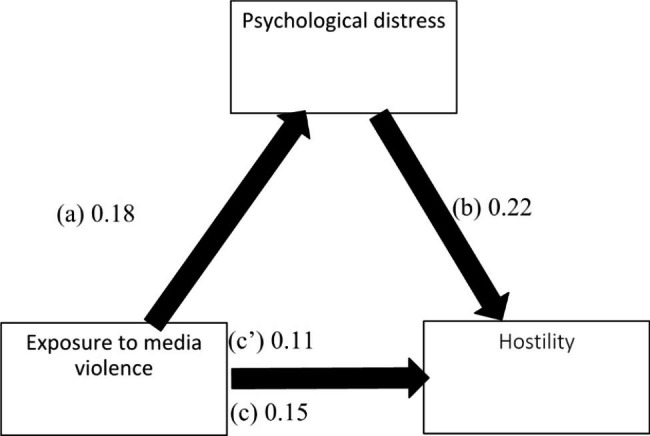
(**a**) Relation between exposure to media violence and psychological distress (R^2^ = .061); (**b**) Relation between psychological distress and hostility (R^2^ = .316); (**c**) Total effect of exposure to media violence and hostility (R^2^ = .127); (c’) Direct effect of exposure to media violence and hostility. Numbers are displayed as regression coefficients (standard error). ***p < .001

**Fig. 4:**
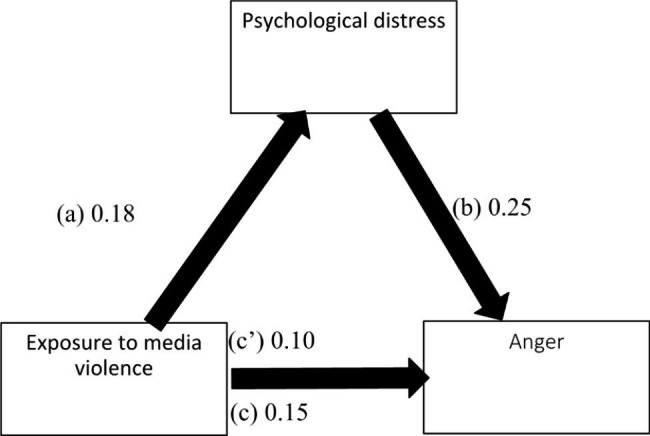
(**a**) Relation between exposure to media violence and psychological distress (R^2^ = .061); (**b**) Relation between psychological distress and anger (R^2^ = .378); (**c**) Total effect of exposure to media violence and anger (R^2^ = .122); (c’) Direct effect of exposure to media violence and anger. Numbers are displayed as regression coefficients (standard error). ***p < .001

The results of the mediation analysis were adjusted over the following variables: age, sex, education, perceived social competence and loneliness. Psychological distress partially mediated the association between exposure to media violence and all forms of aggression (physical, verbal, hostility and anger) (Table 5). Higher exposure to media violence was significantly associated with more psychological distress, which was significantly associated with higher levels of all types of aggression. Moreover, higher exposure to media violence was significantly associated with higher levels of all types of aggression (Figs. 1, 2, 3 and 4).

**Table 5:** Mediation analyses’ results, taking exposure to media violence as the independent variable, psychological distress as the mediator and aggression scores as dependent variables

	Direct effect			Indirect effect		
	Beta	SE	*P*	Beta	Boot SE	Boot CI
Physical aggression	0.01	0.001	< 0.001	0.001	0.0004	0.0004; 0.002*
Verbal aggression	0.07	0.02	< 0.001	0.02	0.01	0.01; 0.03*
Hostility	0.11	0.02	< 0.001	0.04	0.01	0.02; 0.06*
Anger	0.10	0.02	< 0.001	0.05	0.01	0.02; 0.07*

*indicates significant mediation. Direct effect refers to the direct association between exposure to media violenceand aggression without the effect of the mediator, whereas the indirect effect refers to the same association through the mediator (psychological distress)

## Discussion

Media violence is a common theme in Lebanon. To our knowledge, this is the first study of its kind to explore the relationship between media violence and aggression subtypes in this country. First, higher exposure to media violence was associated with all four aggression subtypes while psychological distress was associated with verbal aggression, hostility, and anger but not physical aggression. Second, higher BMI was associated with higher physical aggression and hostility while increased loneliness and decreased social competence were associated with higher verbal aggression and anger. Finally, confirming our hypothesis, psychological distress partially mediated the association between media violence and all aggression subtypes.

### The relationship between exposure to media violence and aggression

We found that higher exposure to media violence is positively associated with an increase in all forms of aggression. This is in line with current literature where violent media positively correlated with aggression [71], higher probability of hostility appraisals [72], and aggressive cognitive responses [73]. Studies also elucidated similar associations amongst children and adolescents [74, 75], including higher media exposure predicting higher verbal and physical aggression in children [76]. These findings could be explained by desensitization to aggressive stimuli caused by repetitive exposure to violent media [17]. Indeed, it is known that repeated exposure to a certain stimulus leads to loss of interest and reactivity to that stimulus. More particularly, recent literature provides us with some evidence that exposure to media violence can make people less empathetic and less physiologically aroused by portrayals of violence in both the short-term and over time [77]. In fact, desensitization, along with observational learning, have been proposed as processes by which media violence leads to aggression [18]. On the other hand, it was also revealed that exposure to violent media is associated with executive control impairments, which predicts high levels of impulsive aggression and hostility [78].

### Body Mass Index as a correlate of aggression

Higher BMI predicted higher physical aggression and hostility in our sample of adults. Similar results in children and adolescents were found. A 2017 meta-analysis by Tso et al. found children and adolescents who were overweight or obese were more likely to be physically aggressive [23]. Authors suggested these findings could be explained through common theoretical determinants such as socio-economic status, peer rejection, mental health issues, and problems in self-regulation. It is likely that these same determinants are common between adults and children as well as also underpin the relationship between higher BMI and hostility in our sample. The same result was found in other studies [79, 80], however, one study on adult male offenders found the opposite relationship [24].

### Loneliness and social competence as correlates of aggression

We found that loneliness is associated with anger and verbal aggression. This relationship has been previously established [81]. Indeed, loneliness was shown be associated with a multitude of psychological variables including anger, anxiety, and pessimism even when variables such as personality traits are controlled for [82]. According to Brinker et al., the experience of loneliness seems to be associated with an increase in aggressive behavioral tendencies or a lack of inhibition. Moreover, some lonely individuals may resort to harmful and destructive strategies as maladaptive means of coping with their loneliness, thereby projecting their anger onto others. Yavuzer et al. [83] supported this hypothesis and revealed that loneliness also played a mediating role between self-theory (one’s perceptions of one’s self) and aggressive behavior.

In contrast, perceived social competence was associated with less anger and less verbal aggression in our sample. This is in line with previous findings [83, 84]. When participants were exposed to aspects of social competence such as leadership perception, there was less perceived anger. In fact, exposure to positive behavior helps in better management of one’s anger and aggressive thoughts.

### The relationship between psychological distress and aggression

As previously discussed, there is a robust association between mental distress and aggression in line with our findings [39, 40]. It is important to note that mental distress increases the relative risk of aggression but the absolute risk is low [42]. In fact, mental distress is not sufficient for aggression to occur as a range of individual and psychosocial factors play a key role [85]. One particular consideration is trauma. Post-Traumatic Stress Disorder (PTSD) can develop through exposure to media coverage of a traumatic event [86]. In fact, trauma exposure and PTSD is linked with both higher rates of aggression [87] as well as psychological distress [88]. As such, violent media that are traumatic to the observer could be a subset of media violence of particular importance in the link between psychological distress and aggression.

### Mediating effect of psychological distress in the association between exposure to media violence and aggression

As expected, psychological distress partially and positively mediated the association between violent media exposure and all forms of aggression. These findings are in agreement with the literature consistently reporting a positive association between exposure to violent media and psychological distress [47][46, 49][89], as well as previous data supporting that distress is closely and positively related to aggression [45]. Immediate effects from exposure to violent media are likely related to the activation of the fight-or-flight response [47, 90]. Long-term possibly lasting effects probably have more complex explanations. For one, violent media is associated with common psychopathological concepts like desensitization, more antisocial behaviors, and less empathy [13]. For another, it is possible that media violence and distress can be part of a positive-feedback loop, especially trauma-related content [49] such as the one in Lebanon [51, 52]. Research into the matter is still needed. Finally, Hasan et al. found that violent games increase stress [46] and suggested that, through this stress increase, violent games can lead to an increase in aggressive behaviors. This is similar to our finding regarding psychological distress as a mediator.

We interpret our mediation results within the available evidence that such effects are likely short term in adults with less long-term impact, as opposed to what is seen in children [15]. We propose that distress could be involved in two psychological processes related to the short-term translation of media violence to aggressive behaviors: priming and arousal. It is worthy to note that long-term processes are likely involved as well and that repeated short-term processes following violent media exposure may lead to cumulative effects [72]. Priming is an inherent linking process between an external stimulus (here, media violence) and a psychological process (here, the notion of aggression) which makes behaviors related to said process more likely (here, aggressive behavior) [18]. In our sample, priming can possibly explain, at least partially, the direct effect of media violence on aggression. Herein, we suggest that psychological distress indirectly reinforces this priming effect. It is possible that, in those with risk factors, media violence is a potent stimulus that triggers a cascade of events in the brain leading to psychological distress and subsequently, makes aggression much more likely. The plausibility of this hypothesis is supported by the common and interrelated neurobiological pathways (e.g. limbic system and amygdala) between psychopathologies (and by extension, aspects of psychological distress) and aggression [39, 91]. As such, psychological distress could increase the chances of aggressive behavior resulting from the primed stimulus of media violence. In sum, media violence is more likely to result in aggression when psychological distress indirectly reinforces the initial primed effect of media violence. Further research is needed to support this hypothesis and explore any direct links or dependencies between the priming process and psychological distress.

Another likely process involved is arousal. Media violence can provoke psychological and physiological arousal through emotions like anger and fear which could lead to disinhibition and/or provocation transfer, resulting in aggression [18, 92]. Additionally, it is no surprise that arousal is related to psychological distress [90, 93]. In fact, it is possible they reflect certain common underlying functions such as attending to threats [90, 94]. Therefore, it is intuitive to assume that psychological distress works similarly to arousal in mediating between media violence and aggression. Moreover, psychological distress, through trans-diagnostic entities like emotional dysregulation [95], impulsivity, and compulsivity [96], could underpin this mediation. In fact, one study found that emotional dysregulation plays a key role in mediating between negative affect and physical aggression [97]. Another study implicated impulsivity and sensation-seeking as predictors of aggressive behavior [98]. This is also reflected in current therapeutic modalities for aggression. For example, Dialectical Behavioral Therapy, a therapy initially devised to treat emotional dysregulation and maladaptive behaviors in borderline personality disorder, has shown promise in the treatment of anger and aggressive behavior [99]. Nothing conclusive can be drawn but it would be valuable for future research to explore what underlying aspects of psychological distress are implicated in mediating between media violence and aggression. In fact, one could conceptualize that those different underlying entities mediate from media violence towards different subtypes of aggression with different effect powers (e.g. impulsivity could mediate more towards behavioral aggression while emotional dysregulation could mediate more towards anger). Future studies are needed on the matter.

### Clinical implications

As rates of aggressive behaviors in adults are becoming alarming, it is becoming increasingly necessary to identify sources of such behaviors. Our results imply that safeguards are needed against exposure to violent media even in adult populations and especially, in the context of Lebanon. Using algorithms to identify violent media content on social media platforms may prove beneficial in limiting such exposure. Moreover, responsible media reporting on violent events is a must. For instance, it is likely helpful to avoid unduly repeating clips of violent events, such as clips from the Beirut Blast, shootings, and robberies. Even though exposure to violent media is a modifiable risk factor, interventions to decrease aggression require a multi-layered approach, including determining individuals at risk. As such, key risk factors like loneliness and psychological distress need to be equally mitigated. Additionally, in contexts where limiting exposure to violent media is unrealistic, likely the current case of Lebanon, clinicians are advised to employ therapeutic modalities aiming to reduce psychological distress, the mediator from violent media to aggression. Access to affordable and adequate mental health services is a prerequisite. Finally, future efforts should be directed towards what trans-diagnostic entities likely underpin the mediation effect from media violence to aggression which could lead to more targeted management strategies.

### Limitations

An information bias is present as in all cross-sectional studies since participants tend to over or underrate their symptoms. A selection bias is present because of the convenient sampling method used in this study and because of the unknown refusal rate for participation, thus, our results might not be generalizable. Most of our young adult sample is female, single, and university educated. As such, this study is not well-suited to elucidate differences related sex nor socio-economic status in the studied variables. Moreover, confounding bias is present since not all factors associated with aggression were considered in this study. The C-ME scale is not validated in Lebanon; results should be consequently interpreted with caution. Finally, the study was a cross-sectional design, thus causal relations between the studied variables could not be explored.

## Conclusion

Psychological distress partially mediated the association between exposure to violent media and all subtypes of aggression in a sample of Lebanese adults. This could be explained by the reinforcement of the priming process of violent media as well as heightened arousal of the individual. Moreover, the higher the violent media exposure, the higher the aggression. This re-opens the discussion on whether violent media should be considered a public health hazard, notably in sociopolitical contexts like Lebanon, where consumption of violent media is the rule. As such, more research is needed on the effects of media violence on adults, especially when such exposure is contextually common.

## Data Availability

All data generated or analyzed during this study are not publicly available due to the restrictions by the ethics committee. The dataset supporting the conclusions is available upon request to the corresponding author.

## Abbreviations

BMI: Body Mass Index
COVID-19: Coronavirus Disease 2019
HCI: Household Crowding Index
C-ME: Content-Based Media Exposure Scale
BPAQ-SF: Buss Perry Agression Questionnaire
DASS-8: Depression, Anxiety, Stress Scale
PSCS: Perceived Social Competence Scale
SPSS: Statistical Package for the Social Sciences
